# Editorial: Breaking the biofilm barrier: analysis of molecular mechanisms underlying biofilm formation and identification of novel antimicrobial approaches

**DOI:** 10.3389/fmicb.2026.1856687

**Published:** 2026-05-19

**Authors:** Eliana De Gregorio, Emmanuelle Dé, Arianna Pompilio, Asad U. Khan, Raffaele Zarrilli

**Affiliations:** 1 Department of Molecular Medicine and Medical Biotechnology, University of Naples Federico II, Naples, Italy; 2 UMR 6570 CNRS, Rouen Normandy University, Rouen, France; 3 Department of Medical, Oral and Biotechnological Sciences, “G. d'Annunzio” University of Chieti-Pescara, Chieti, Italy; 4 Antimicrobial Resistance Lab, Interdisciplinary Biotechnology Unit, Aligarh Muslim University, Aligarh, India; 5 Department of Public Health, University of Naples Federico II, Naples, Italy

**Keywords:** antibiofilm agents, antibiofilm strategies, biofilm eradication, efflux pumps, regulation of biofilm growth

Microbial biofilms represent a major challenge in public health and infection control. This Research Topic brings together recent original updates on the processes underlying biofilm formation and on the novel antimicrobial approaches to effectively disrupt and eradicate biofilms.

Four articles of this topics investigated the mechanisms involved the development, maintenance, and resistance/tolerance of biofilms.

Muturi et al. analyzed the role of biofilm formation in chronic carriage of *Salmonella typhi*, particularly in individuals with gallstones in typhoid-endemic settings. Analysis of 22 clinical isolates (genotype H58) revealed that multidrug resistance (MDR) genes were restricted to specific sub-lineages, suggesting heterogeneity in resistance profiles. The isolates demonstrated the ability to form biofilms under gallbladder-like conditions, particularly on cholesterol-coated surfaces mimicking gallstones. Interestingly, non-MDR strains exhibited significantly stronger biofilm-forming capacity compared to MDR strains, suggesting a potential evolutionary trade-off between antimicrobial resistance and biofilm fitness. The study also identified specific genetic mutations associated with persistence, including a missense mutation in the *treB* gene (A383T) and alterations in Vi capsular polysaccharide genes such as *tviE*. These mutations were observed predominantly in isolates collected after clinical recovery, suggesting their role in adaptation to the gallbladder environment and long-term colonization.

The study performed by Wang S. et al. explored the role of the sigma factor FliA in modulating the antibacterial effectiveness of plantaricin BM-1 against *Escherichia coli* K-12. It found that FliA influences bacterial susceptibility through the LuxS/AI-2 quorum-sensing system, impacting biofilm development and responses to antimicrobial peptides. Disrupting this pathway changed biofilm behavior and decreased sensitivity to plantaricin BM-1. These results offer new understanding of the complex interactions among quorum sensing, gene regulation, and antimicrobial action, highlighting potential targets for enhancing antibiofilm therapies.

Park et al. analyzed the exposure–response relationship of tigecycline against *Mycobacterium abscessus*. This study used a hollow fiber infection model integrated with PK/PD, which mimicked pulmonary infection conditions and tigecycline lung exposure under intrapulmonary aerosol administration. While high tigecycline exposure resulted in effective bacterial killing, suboptimal dosing led to regrowth and emergence of less susceptible subpopulations. Biofilm formation impaired antibacterial activity by restricting drug penetration and generating concentration gradients that reduced local exposure. This model captured key processes, including dose-dependent killing, resistance development, and biofilm-associated permeability changes. Simulations indicated that substantially higher doses are required to eradicate biofilm-embedded bacteria, particularly under delayed treatment conditions, highlighting key determinants of therapeutic efficacy.

The review by Zhang et al. provided a detailed overview of how *Pseudomonas aeruginosa* causes disease, emphasizing the interactions between virulence factors, quorum sensing, and biofilm development. The authors underlined how these interconnected processes boost bacterial adaptability, persistence, and resistance to antimicrobial therapies. Special focus is given to the combined regulation of virulence and biofilm traits, which increases the pathogenic potential in clinical environments. The work highlights the need to target multiple pathways at once to effectively manage *P. aeruginosa* infections and biofilm formation.

Several published studies explored the development of innovative antimicrobial strategies aimed at effectively preventing biofilm formation or eradicating established biofilms.

The investigation conducted by Li et al. evaluated the potential of probiotic-based interventions in mitigating biofilm-associated infections caused by ST11 carbapenem-resistant *Klebsiella pneumoniae* (CRKP). The authors analyzed a total of 334 ST11 CRKP isolates obtained from multiple hospitals, demonstrating exceptionally high resistance rates (>90%) to diverse classes of antibiotics. Additionally, notable regional variation in capsular serotypes was observed, with K64 predominating in eastern China and K47 being more prevalent in central regions. As over 97% of isolates were capable of producing biofilms, this study investigated the anti-biofilm effects of probiotic bacteria, specifically *Lactobacillus fermentum* and *Lactobacillus gasseri*. Co-culture experiments demonstrated a significant reduction in biofilm biomass (41–58%). Metabolomic analysis demonstrated that *L. fermentum* disrupted purine biosynthesis and aminoacyl-tRNA metabolism, while *L. gasseri* inhibited folic acid synthesis and the phosphotransferase system. In summary, this research highlights the potential of probiotics as adjunct or alternative therapies for combating persistent and resistant bacterial infections.

Yu et al. developed a novel orthodontic appliance by incorporating fluoride sodium (NaF) at 0.5–1.5% into the silicone prior molding, in order to ensure sustained release for approximately 28 days. At concentrations up to 1.5%, NaF did not alter significantly the mechanical properties of the novel silicone appliance material, nor it reduced the viability of cell lines below 85% but it successfully inhibited the biofilm formation and acid production of *Streptococcus mutans* or saliva-derived biofilms. Finally, this new NaF-containing material prevented enamel demineralization, indicating that it may present interesting anti-caries properties.

Three studies examined the synergistic effects of combining different treatments to help break down and remove biofilms.

Migliaccio et al. analyzed the susceptibility of *Acinetobacter baumannii* biofilms to biocides, chlorhexidine (CHX) and benzalkonium (BZK) alone or in combination. These compounds clearly decreased the biofilm formation of reference and multidrug resistant strains at subMIC concentrations. Their combination with the natural stilbenoid resveratrol (RV) demonstrated a pronounced synergistic effect, resulting in complete inhibition of biofilm formation on solid surfaces and a substantial reduction (approximately 70–80%) in biofilm development at the air–liquid interface. Furthermore, CHX–BZK–RV combinations exhibited an additive effect against preformed biofilms at concentrations that were non-cytotoxic. The activity of RV within these combinations appears to be mediated through the downregulation of the *adeB* gene, a key component of the Resistance Nodulation Division efflux pump system. Collectively, these findings suggest that CHX–BZK–RV combinations hold promise for incorporation into novel and effective anti-biofilm formulations.

The synergistic antibacterial and anti-biofilm efficacy of the ceftazidime–avibactam (CZA) and aztreonam (ATM) combination against carbapenem-resistant *Klebsiella pneumoniae* (CRKP) has also been systematically investigated by Wang G. et al. A total of 150 clinical CRKP isolates were analyzed, representing diverse resistance mechanisms, including the presence of carbapenemase genes such as *bla*_KPC-2_ and *bla*_NDM-1_. The results demonstrated a strong synergistic effect of CZA and ATM, particularly against strains producing *bla*_KPC-2_. The combination therapy significantly inhibited biofilm development. Furthermore, quantitative PCR analysis revealed downregulation of key biofilm-associated genes, including *luxS, mrkA, wbbM, pgaA*, and *wzm*. In conclusion, this study highlights the importance of combination therapy against CRKP and emphasizes the need to consider biofilm inhibition as a key component of antimicrobial treatment in chronic and device-associated infections.

Research combining perillaldehyde with domiphen showed enhanced antibacterial and antibiofilm effects against *Staphylococcus aureus* and *Escherichia coli* (Qiao et al.). This combination greatly improved bactericidal outcomes compared to using each agent alone, effectively preventing biofilm formation and breaking down existing biofilms. The synergy probably resulted from increased membrane permeability and disruption of bacterial physiological functions. These results suggest that pairing natural compounds with traditional antiseptics could be a promising approach to address biofilm-related infections.

Several of the published manuscripts analyzed the properties of natural compounds on the inhibition of biofilm growth and on the eradication of preformed biofilms.

Zhao et al. reported that the sesamin-derived arylnaphthalene lignan JR20 exhibited strong antibacterial activity against methicillin-resistant *Staphylococcus aureus* (MRSA), with an IC_50_ of 20.88 μg/mL, and effectively inhibited biofilm formation and disrupted established biofilms. Confocal and electron microscopy analyses revealed pronounced bacterial cell wall damage and morphological alterations, accompanied by a concentration-dependent depletion of intracellular ATP, indicating metabolic impairment as a central mechanism of action. Molecular docking analyses suggested an allosteric binding to PBP2a protein, potentially impairing cell wall synthesis. Furthermore, *in vivo* murine models confirmed its anti-MRSA efficacy and reduced MRSA-induced inflammation and neutrophil infiltration in murine liver and spleen. Despite moderate cytotoxicity, JR20 demonstrates significant anti-biofilm and antibacterial potential, representing a promising lead for novel drug-resistant bacterial therapeutics.

The study performed by Shen et al. identified RmmLII, a novel N-acylhomoserine lactonase from *Tritonibacter mobilis* YJ3, as a quorum-quenching enzyme that targets bacterial communication. RmmLII, belonging to the phosphotriesterase family, hydrolyzed a broad range of AHL signaling molecules (C6–C14), with preference for long-chain and 3-oxo-substituted AHLs, thereby disrupting quorum sensing in *Pseudomonas aeruginosa* PAO1. *In vitro*, it significantly inhibited quorum sensing–regulated virulence factors in PAO1, including proteases, pyocyanin, rhamnolipids, motility, and biofilm formation. In a murine lung infection model, RmmLII reduced bacterial burden, suppressed pro-inflammatory cytokines (IL-1β, IL-6, TNF-α), and enhanced survival rates. These findings highlight RmmLII as a promising anti-virulence strategy to control biofilm-associated infections.

Ahmad et al. explored the effects of b-N-acetylglucosaminidase (NAGase), an enzyme derived from jack beans, to prevent biofilm formation and disrupt established biofilms in *Staphylococcus aureus*, either alone or in combination with thymoquinone (TQ) extracted from *Nigella sativa* seeds. Treatment with NAGase and TQ reduced *S. aureus* preformed biofilms by 61.8%−73.8%. Computer simulations revealed that the TQ ligand was a potent inhibitor of quorum sensing (QS) molecules. Also, NAGase, alone or in combination with TQ, decreased the expression of the *atl, icaA*, and *agr* QS genes. In conclusion, the study indicates that the combination of TQ and NAGase is a promising antibiofilm approach against *S. aureus*.

Yin et al. studied the effects of a cinnamaldehyde nanoemulsion (Cin-NE) combined with rhamnolipid (RHL) on methicillin-resistant *Staphylococcus aureus* (MRSA) biofilm formation. CIN-NE, either alone or in combination with RHL, inhibited MRSA biofilm formation by 65.5% and 66.9%, respectively, and eradicated MRSA preformed biofilm by 76.3% and 84.6%, respectively. In contrast, CIN eradicated MRSA preformed biofilms by only 24.1%. Moreover, Cin-RHL-NE inhibited the inflammation induced by MRSA biofilms implanted subcutaneously into the back of BALB/c mice. In conclusion, this study identifies the Cin-RHL-NE formulation as a promising candidate for biofilm eradication, acting both *in vitro* and *in vivo*.

The study performed by Taylor et al. investigated the antibiofilm and antivirulence properties of 4-ethoxybenzoic acid molecule, a natural compound isolated from the East african shrub *Rhamnus prinoides*. Its use at 0.8 mg/mL decreased up to 88% the biofilm formed by *Staphylococcus aureus* ATCC 65383 but also drastically reduced the production of several virulence factors like the α-hemolysin, the serine protease C, the staphyloxanthin, decreasing consequently the *Staphylococcus* hemolysis activity up to 87%. *In silico* docking analysis suggested that this molecule could act via its interaction with more than 25 proteins, including several transcriptional regulators or sensor kinases, like SaeS, essential to the pathogen virulence.

The study by Brożyna et al. analyzed the antibiofilm activity of *Rosmarinus officinalis* L. and *Thymus vulgaris* L. essential oils (EO) against a panel of clinical *Staphylococcus aureus* isolates obtained from non-healing wounds. Thymus EO, which contains a higher proportion of phenolic compounds like thymol and carvacrol, exhibited higher antibiofilm activity than rosemary EO, dominated by oxygenated monoterpenes such as 1,8-cineole and α-pinene. This suggests that thymus EO may interact more strongly with bacterial membranes and disrupt biofilm metabolic processes, while rosemary oil may have weaker interactions with dense matrix structures. Also, liquid forms of rosemary EO displayed significantly higher antibiofilm activity than volatile forms of rosemary EO, suggesting that the antibiofilm activity of essential oils is influenced by the delivery method.

In summary, multiple mechanisms regulate growth and persistence of microbial biofilms. The identification of novel antimicrobial compounds and therapeutic regimens is important to inhibit biofilm growth and eradicate preformed biofilms.

